# Predictive value of the electrocardiogram exercise stress test for the presence or absence of left main disease

**DOI:** 10.3389/fcvm.2025.1675602

**Published:** 2025-10-01

**Authors:** Saverio Tremamunno, Nello Cambise, Angelo Giuseppe Marino, Fabio De Benedetto, Ludovica Lenci, Cristina Aurigemma, Carlo Trani, Francesco Burzotta, Gaetano Antonio Lanza

**Affiliations:** ^1^ Department of Cardiovascular Sciences, Fondazione Policlinico Universitario A. Gemelli IRCCS, Roma, Italy; ^2^ Department of Cardiovascular and Pneumological Sciences, Università Cattolica del Sacro Cuore, Rome, Italy

**Keywords:** coronary artery disease, left main disease, electrocardiogram exercise stress test, invasive coronary angiography, negative predictive value

## Abstract

**Background:**

The ability of the electrocardiogram exercise stress test (ECG-EST) in excluding the presence of left main (LM) coronary artery disease (CAD) has been poorly investigated.

**Methods:**

We retrospectively selected patients who underwent both ECG-EST and elective invasive coronary angiography (ICA) at our Institution between January 2018 and December 2023 due to angina pain suspected of obstructive CAD. Preventively defined individual and combined ECG-EST variables suggesting no/mild myocardial ischemia were assessed as predictors of the absence of LM disease. Some ECG-EST variables suggesting extensive/severe myocardial ischemia were instead assessed as predictors of the presence of LM disease, defined as a stenosis ≥50% of the left main artery.

**Results:**

Overall, 515 patients were included (age 66.2 ± 11 years; 74% men). LM disease at ICA was found in 26 patients (5%). Individual and combined ECG-EST variables showed low positive predictive values for LM-CAD [maximum 15% for a combination of ST-segment depression (STD) in ≥ 5 leads and ECG-EST duration <360 s]. The negative predictive value, however, was very high for some combined ECG-EST variables. Very low risk of LM disease (≤2.5%) was particularly shown in patients with peak heart rate (HR) ≥ 75% of maximal predicted HR for age and STD < 2 mm (prevalence 63.1%; risk 2.2%) and peak HR ≥85% of maximal predicted HR for age and maximal STD < 2 mm (prevalence 46.2%; risk 2.5%).

**Conclusions:**

Among patients with angina chest pain suspected of obstructive CAD, ECG-EST results can reliably identify those at very low risk of LM disease at coronary angiography.

## 1 Introduction

Randomized clinical trials in the 1980s showed that, among patients with suspected or ascertained obstructive coronary artery disease (CAD), coronary artery bypass graft surgery consistently improved survival only in patients with left main (LM) CAD or those with three-vessel disease CAD who also had impaired left ventricular function (1, 2).

Importantly, while subsequent randomized clinical trials confirmed the lack of any significant benefit on survival of coronary revascularization interventions, compared to optimal medical therapy, in patients with stable CAD but no evidence of LM disease (3–5), percutaneous coronary intervention (PCI) was found to provide similar survival rates to bypass surgery in several subgroups of patients with LM disease (6–8). According to these results, most patients with suspected or known CAD could be treated by optimal medical therapy only, but exclusion of LM disease and impaired left ventricular function would be mandatory.

Several studies investigated whether LM disease might be identified through electrocardiogram exercise stress test (ECG-EST), showing that some abnormal ECG-EST results, particularly, stress-induced ST-segment elevation (STE) in lead aVR and/or V1, ST-segment depression (STD) in five or more leads, and a greater magnitude of STD, may predict the presence of LM disease (9–11). However, according to the previously mentioned results from clinical trials, ruling out LM disease in individual patients would be just as important as identifying it, since it would suggest that conservative medical treatment might be sufficient for their management.

Thus, in this study, we specifically aimed to investigate, in a population of patients referred to undergo a first ECG-EST for a suspected angina chest pain, whether and how accurately ECG-EST results may reliably exclude the presence of LM disease. To this scope, we preventively chose a series of ECG-EST variables and combinations of variables suggesting either no/mild myocardial ischemia and assessed their relationship with the absence of LM disease at invasive coronary angiography (ICA). At the same time, however, we also assessed the relation with LM disease of preventively defined ECG-EST variables indicating severe/extensive myocardial ischemia.

## 2 Methods

We retrospectively included in this study consecutive clinically stable patients who underwent both ECG-EST and elective ICA at our Department of Cardiovascular Sciences, Policlinico Universitario A. Gemelli IRCCS, Rome, between 1 January 2018 and 31 December 2023, due to suspected obstructive CAD.

The patients were excluded when any of the following conditions were present: (1) ICA performed after >6 months from the ECG-EST; (2) ECG abnormalities preventing reliable assessment of ST-segment changes during ECG-EST (e.g., atrial fibrillation, intraventricular conduction disorders, pacemaker rhythm, and significant ST-segment abnormalities at baseline); (3) use of an ECG-EST protocol different from standard treadmill Bruce protocol; or (4) any previous history of CAD or coronary angiography. The main clinical data of the patients were obtained from the institutional database of our hospital.

### 2.1 Exercise stress test

All patients underwent treadmill ECG-EST following a standard symptom- and sign-limited Bruce protocol. Three ECG leads (DII, V2, and V5) were continuously monitored throughout the test, and up-to-date averaged QRS complexes from all ECG leads continuously displayed on the screen. Criteria for stopping the test included physical exhaustion, increasing angina severity (Borg scale >6) (12), relevant clinical events (e.g., dyspnea, severe arrhythmias, hypotension), and STD >4 mm in any ECG lead. Blood pressure (BP) and heart rate (HR) were recorded at baseline and at peak EST. A summary report with full ECG-EST and 10 s 12-lead ECG strips recorded at baseline, at the end of each stage, at the time of significant ST-segment changes, at peak EST, every minute in the recovery phase, and when clinically indicated, was digitally stored in a database and available for analysis.

ECG-ESTs were reviewed, and the following findings were obtained: (1) HR and systolic and diastolic BP at baseline and peak exercise; (2) duration of the test; (3) occurrence of STD ≥1 mm in each lead, except aVR; (4) maximal STD; (5) number of leads showing STD ≥1 mm; (6) occurrence of symptoms (angina, dyspnea, etc.).

Two investigators independently reviewed ECG-ESTs, and discrepancies were solved by consensus and supervision of a third investigator. ECG-EST was considered positive for myocardial ischemia when a horizontal or downsloping STD ≥1 mm at 60–80 ms from the J-point was detected in at least two contiguous leads, but not aVR. Furthermore, EST was considered maximal when peak HR was above 75% of maximal predicted HR for age, calculated according to Fox's formula: maximal HR = 220 − age (in years) (13).

### 2.2 ECG-EST variables predictive for the presence/absence of LM disease

Based on the results of our previous study (10), we assessed the association with LM disease of the following preventively established individual and combined variables suggesting severe and/or extensive myocardial ischemia: (1) STD involving ≥5 ECG leads; (2) STD ≥2 mm; (3) STD involving ≥5 ECG leads and ECG-EST duration <360 s; and (4) STD >2 mm and ECG-EST duration <360 s. Furthermore, the relationship between LM disease and ST-segment elevation in lead aVR was also assessed (14–16).

The following, preventively established ECG-EST variables, suggesting no/mild myocardial ischemia, were instead tested for the absence of LM disease at ICA: (1) negative ECG-EST; (2) ECG-EST duration ≥12 min; (2) ECG-EST duration ≥9 min; (3) ECG-EST duration ≥9 min with negative ECG-EST; (4) peak HR ≥85% of maximal predicted HR for age with negative EST; (5) peak HR ≥75% of maximal predicted HR for age with negative EST; (6) peak HR ≥85% of maximal predicted HR for age and STD < 2 mm; and (7) HR ≥75% of maximal predicted HR for age and STD < 2 mm.

ECG-EST variables were considered to be associated with a very low risk of LM disease when the latter was found in ≤2.5% of patients.

### 2.3 Invasive coronary angiography

ICA was performed within 6 months of EST (usually within 1 month) through radial access, following standard procedures. LM disease was considered to be present when a stenosis of ≥50% of the vessel lumen of the LM coronary artery was detected. Coronary stenoses in the other main epicardial coronary arteries were considered significant if they caused a ≥70% reduction of the vessel lumen or were associated with a fractional flow reserve ≤0.80.

### 2.4 Statistical analyses

All variables assessed in this study showed a distribution not significantly different from normal according to Kolmogorov–Smirnov testing. Thus, continuous variables were compared by analysis of variance, whereas comparisons of proportions were performed using the chi-square test. Data are reported as means with standard deviations and numbers (percentages). Statistical significance was defined as *p* < 0.05. Data were analyzed with SPSS 28.0 statistical software (SPS Statistics, Florence, Italy).

## 3 Results

### 3.1 Characteristics of the population

Overall, among 15,550 ECG-ESTs performed at our exercise stress test laboratory between January 2018 and December 2023, 515 (3.3%) were performed in patients fulfilling the inclusion/exclusion criteria for the study. The main clinical characteristics and angiographic findings of patients eventually included in the study are summarized in Table 1.

**Table 1:** Main clinical characteristics of patients included in the study.

Number of patients	515
Age (years)	66.2 ± 10.6
Sex (M)	381 (74.0%)
Body mass index (kg/m^2^)	26.5 ± 4.6
Cardiovascular risk factors	
Hypertension	402 (78.1%)
Diabetes	136 (26.4%)
Hypercholesterolemia	238 (46.2%)
Smoking	267 (51.8%)
Beta-blocker therapy	226 (43.9%)
Number of diseased vessels at ICA^a^	
0	238 (46.2%)
1	147 (28.5%)
2	59 (11.5%)
3	71 (13.8%)

CAD, coronary artery disease; ICA, invasive coronary angiography; PCI, percutaneous coronary intervention.

^a^ Number of diseased coronary vessels other than the left main artery.

The study population included 381 men (74%) and 134 women (26%), with a mean age was 66.2 ± 11 years. The most prevalent cardiovascular risk factor was hypertension (78.1%), and diabetes was present in 26.4% of patients. Approximately half of patients (43.9%) were taking beta-blocker therapy, either bisoprolol (*n* = 156, 69%) or metoprolol (*n* = 73, 31%). A low dose of beta-blockers (i.e., ≤ 5 mg/day of bisoprolol or ≤100 mg/day of metoprolol) was taken by 97% of patients. Furthermore, 23.5% of patients were taking calcium-channel blockers, and 3.9% were taking ranolazine.

Overall, 26 patients (5%) were found to have LM disease at ICA. Intracoronary physiological or imaging assessment to confirm the hemodynamic relevance of left main stenosis was performed in only three (12%) patients: fractional flow reserve, optical coherence tomography, and intravascular ultrasound (IVUS) imaging. Treatment of patients with LM disease included coronary artery bypass grafting in 13 patients (50%) and percutaneous coronary intervention in 10 (38%), whereas 3 patients (12%) were managed with medical therapy alone.

Besides LM disease, ICA documented obstructive CAD in 277 patients (53.8%), and one-, two-, and three-vessel CAD were found in 28.5%, 11.6%, and 13.8% of patients, respectively.

### 3.2 Clinical/ECG-EST findings and LM disease

The main clinical and angiographic findings, as well as the primary results of the ECG-EST, for patients with and without LM disease are summarized in Tables 2 and 3, respectively. As shown, among the clinical characteristics, only male gender (*p* = 0.029) was significantly associated with LM disease.

**Table 2:** Main clinical characteristics and angiographic findings of patients with and without left main disease.

	LM disease (*n* = 26)	No LM disease (*n* = 489)	*p*
Age	69.1 ± 10.8	66.1 ± 10.6	0.15
Sex (M)	24 (92.3%)	357 (73.0%)	0.029
Body mass index (kg/m^2^)	26.1 ± 2.3	26.5 ± 4.7	0.64
Cardiovascular risk factors			
Hypertension	21 (80.8%)	381 (77.9%)	0,73
Diabetes	9 (36.6%)	127 (26.0%)	0.33
Hypercholesterolemia	11 (42.3%)	227 (46.4%)	0.68
Smoking	17 (65.4%)	250 (51.1%)	0.16
Beta-blocker therapy	15 (57.7%)	211 (43.1%)	0.14
Number of diseased vessels at ICA^a^			<0.001
0	1 (3.8%)	237 (48.5%)	
1	7 (26.9%)	140 (28.6%)	
2	7 (26.9%)	52 (10.6%)	
3	11 (42.3%)	60 (12.3%)	

CAD, coronary artery disease; ICA, invasive coronary angiography; PCI, percutaneous coronary intervention.

^a^ Number of diseased coronary vessels other than the left main artery.

**Table 3:** Main exercise stress test results.

	LM disease (*n* = 26)	No LM disease (*n* = 489)	*p*
Baseline			
Heart rate (bpm)	74 ± 12	76 ± 13	0.41
Systolic BP (mmHg)	131 ± 18	131 ± 17	0.92
Diastolic BP (mmHg)	79 ± 10	79 ± 9	0.99
Peak EST			
Heart rate (bpm)	130 ± 18	139 ± 20	0.02
% heart rate max	86 ± 11	90 ± 12	0.14
Systolic BP (mmHg)	173 ± 25	171 ± 26	0.60
Diastolic BP (mmHg)	92 ± 14	90 ± 12	0.35
EST duration (s)	394 ± 135	417 ± 158	0.46
Ischemic ECG changes			
Positive EST	23 (88.5%)	354 (72.4%)	0.07
No of leads with STD	3.08 ± 2.0	2.42 ± 1.8	0.07
No. of patients with STD in ≥5 leads	6 (23.1%)	49 (10.0%)	0.036
Max STD (mm)^a^	1.64 ± 1.1	1.17 ± 0.9	0.02
STD ≥2 mm	15 (57.7%)	129 (26.4%)	<0.001
STE in aVR	1 (3.8%)	15 (3.1%)	0.82
Other EST findings			
Angina	9 (34.6%)	62 (12.7%)	0.002
Dyspnea	1 (3.8%)	8 (1.6%)	0.40
EST-related VAs	9 (34.6%)	160 (32.8%)	0.59
Recovery VAs	6 (23.1%)	119 (24.5%)	0.70

BP, blood pressure; EST, exercise stress test; STD, ST-segment depression; STE, ST-segment elevation; VAs, ventricular arrhythmias.

^a^ In patients with positive ECG-EST.

Compared with patients without, those with LM disease showed only a tendency to a higher rate of positive ECG-EST (*p* = 0.07), but more frequently showed STD in ≥5 ECG leads (*p* = 0.036) and STD ≥2 mm (*p* < 0.001). Furthermore, maximal STD level during ECG-EST was significantly higher in patients with vs. those without LM disease (*p* = 0.02). Finally, patients with LM disease reported a higher rate of angina (*p* = 0.002). Of note, all but one of the LM disease patients (96.2%) showed a significant stenosis in at least one other coronary artery vessel.

### 3.3 ECG-EST variables and presence/absence of LM disease

The ability of ECG-EST variables selected for predicting the presence of LM disease is summarized in Table 4. As shown, the prevalence and the positive predictive value of these variables were very low.

**Table 4:** ECG-EST predictors for left main disease.

	No. of patients	Prevalence (%)	Positive predictive value (%)
STD in ≥5 leads	55	10.7	10.9
STD ≥2 mm	144	28.0	10.4
STE ≥1 mm in aVR	16	3.1	6.2
STD in ≥5 leads and ECG-EST duration <360 s	20	2.6	15.0
STD in ≥5 leads and HR <75% of maximal HR for age	19	3.7	7.7
STD ≥2 mm and ECG-EST duration <360 s	59	7.7	13.5
STD ≥2 mm and HR <75% of maximal HR for age	57	11.1	12.3

ECG-EST, exercise stress test; HR, heart rate; STD, ST-segment depression; STE, ST elevation.

**Figure 1:**
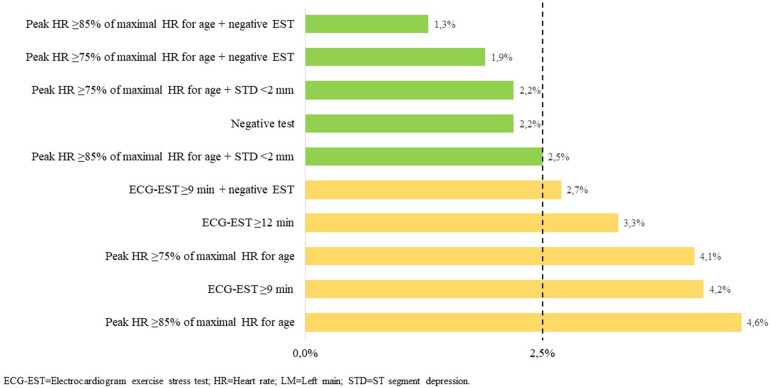
Risk of LM disease of individual and combined ECG-EST variables. Very low risk of LM disease is set at ≤2.5%.

The negative predictive value for LM disease of selected individual and combined ECG-EST variables is summarized in Table 5 and partially shown also in Figure 1.

**Table 5:** Predictive value for the absence of left main disease of individual and combined ECG-EST variables.

	No. of patients	Prevalence (%)	Negative predictive value (%)	Risk of LM disease (%)
Negative test	138	26.8	97,8	2.2
ECG-EST ≥12 min	16	3.1	96.7	3.3
ECG-EST ≥9 min	143	27.8	95.8	4.2
Peak HR ≥75% of maximal HR for age	462	89.7	95.9	4.1
Peak HR ≥85% of maximal HR for age	346	67.2	95.4	4.6
ECG-EST ≥9 min + negative EST	37	7.2	97.3	2.7
Peak HR ≥75% of maximal HR for age + negative EST	108	21.0	98.1	1.9
Peak HR ≥75% of maximal HR for age + STD < 2 mm	325	63.1	97.8	2.2
Peak HR ≥85% of maximal HR for age + negative EST	77	15.0	98.7	1.3
Peak HR ≥85% of maximal HR for age + STD < 2 mm	238	46.2	97.5	2.5

ECG-EST, exercise stress test; HR, heart rate; STD, ST-segment depression; STE, ST elevation.

All considered, the ECG-EST variables were associated with a very low risk of LM disease. A negative ECG-EST (prevalence 26.8%) was associated with only 2.2% of LM disease, whereas the lowest risk (1.3% only) was found in those who had a negative test, achieving 85% or more of the theoretical maximal HR for age (prevalence 15%). A very low risk of LM disease (2.2%) was particularly detected in the subgroup of patients who achieved ≥75% of maximal HR for age and had STD <2 mm, which included 63.1% of patients.

## 4 Discussion

Our data show that, among patients referred for an initial assessment of angina pain suspected of CAD, several ECG-EST results may identify patients with a very low risk (≤2.5%) of LM disease. ECG-EST results, on the other hand, showed a very low predictive value for the presence of the disease.

The present evidence from clinical trials is that, among clinically stable patients with obstructive CAD, coronary revascularization by surgical intervention (CABG) results in improvement of long-term prognosis only when LM disease or three-vessel CAD (with a reduced left ventricular function) is found at coronary angiography (1, 2). However, PCI has been shown to result in similar long-term outcomes compared with CABG in some subgroups of patients with LM disease, particularly non-diabetic patients and those with isolated LM disease or SYNTAX score <32 (6–8).

On the other hand, large clinical trials have demonstrated that, among patients with normal left ventricular function and no LM disease, coronary revascularization does not result in significant clinical advantages compared with optimal medical therapy (3–5). Thus, patients in whom LM disease can reliably be excluded by non-invasive tests might safely be treated with medical therapy and avoid invasive assessment.

In this study, we show that ECG-EST is valuable for this scope. Some individual and combined ECG-EST variables, indeed, were highly predictive for the lack of LM disease being associated with a very low risk of the disease. The lowest rate of LM disease was found in the subgroup of patients who achieved a peak HR ≥85% and had no STD during ECG-EST (only 1.3%), but this subgroup included only 15% of patients. On the other hand, the combination of peak HR ≥75% of the maximal HR predicted for age and ST-segment depression <2 mm was detected in a sizeable proportion of 63.1% of patients and was associated with a 2.2% risk for LM disease. Similarly, the combination of peak HR ≥85% of the maximal HR predicted for age and ST-segment depression <2 mm was detected in 46.2% of patients and was associated with a 2.5% only of the risk of LM disease. In these patients, further tests might, therefore, be safely avoided, even considering that it remains unclear whether prognosis is actually improved by coronary revascularization in the small subgroup of patients with LM disease who do not develop significant signs of myocardial ischemia and show good exercise tolerance during ECG-EST (17).

Our data confirm, on the other hand, that ECG-EST results do not allow for reliable prediction of the presence of LM disease (9–11). In agreement with our previous study (10), extensive (in ≥5 leads) or severe (≥2 mm) ST-segment depression were indeed associated with an increased risk of LM disease, but their positive predictive value for LM disease of ECG-EST remained very low (<20%), thus making these variables of limited utility in the individual patient in clinical practice. Of note, while some data previously reported a high predictive value for LM disease of ST elevation in lead aVR (14–16, 18, 19), the results of the present study confirm our previous observation of the lack of any significant impact on the detection of LM disease of STE in aVR (10), which also showed a very low rate of occurrence in our population.

### 4.1 Limitations of the study

Some limitations of our study should be acknowledged. First, the number of patients with LM disease was rather low, and, therefore, larger studies are desirable to validate the results of this investigation. Second, the indication for coronary angiography was clearly influenced by the ECG-EST results and, for those with a negative test, was probably driven by a high clinical probability of CAD, which suggests that the negative predictive value for LM disease of low-risk ECG-EST is even higher among a less selected population of patients undergoing the test to assess exercise-induced ischemia. Finally, anti-ischemic therapy was not systematically withdrawn before the test, and therefore might have influenced the results of ECG-EST and its relation with the presence/absence of LM disease.

### 4.2 Conclusions

Our data show that, among patients with a history of angina chest pain suspected of obstructive CAD, the results of ECG-EST can identify subgroups of patients at very low risk for LM disease. These patients may be safely managed with medical therapy.

## Data Availability

The raw data supporting the conclusions of this article will be made available by the authors, without undue reservation.
